# Blood lead levels in children after phase-out of leaded gasoline in Kinshasa, the capital of Democratic Republic of Congo (DRC)

**DOI:** 10.1186/0778-7367-71-5

**Published:** 2013-04-04

**Authors:** Joel Tuakuila, Martin Kabamba, Honoré Mata, Gerard Mata

**Affiliations:** 1 Environmental Chemistry, Faculty of Sciences, University of Kinshasa, Kinshasa, Democratic Republic of Congo; 2 Louvain Center for Toxicology and Applied Pharmacology (LTAP), Université catholique de Louvain, Avenue E. Mounier 53, box 52.02.12, Brussels, 1200, Belgium

**Keywords:** Blood lead levels, Leaded gasoline, Children health, Phase out, Kinshasa

## Abstract

**Background:**

The phasing out of lead from gasoline has resulted in a significant decrease in blood lead levels (BLLs) in children during the last two decades. Tetraethyl lead was phased out in DRC in 2009. The objective of this study was to test for reduction in pediatric BLLs in Kinshasa, by comparing BLLs collected in 2011 (2 years after use of leaded gasoline was phased out) to those collected in surveys conducted in 2004 and 2008 by Tuakuila et al. (when leaded gasoline was still used).

**Methods:**

We analyzed BLLs in a total of 100 children under 6 years of age (Mean ± SD: 2.9 ± 1.6 age, 64% boys) using inductively coupled argon plasma mass spectrometry (ICP – MS).

**Results:**

The prevalence of elevated BLLs (≥ 10 μg/dL) in children tested was 63% in 2004 [n = 100, GM (95% CI) = 12.4 μg/dL (11.4 – 13.3)] and 71% in 2008 [(n = 55, GM (95% CI) = 11.2 μg/dL (10.3 – 14.4)]. In the present study, this prevalence was 41%. The average BLLs for the current study population [GM (95% CI) = 8.7 μg/dL (8.0 – 9.5)] was lower than those found by Tuakuila et al. (F = 10.38, p <0.001) as well as the CDC level of concern (10 μ/dL), with 3% of children diagnosed with BLLs ≥ 20 μg/dL.

**Conclusion:**

These results demonstrate a significant success of the public health system in Kinshasa, DRC-achieved by the removal of lead from gasoline. However, with increasing evidence that adverse health effects occur at BLLs < 10 μg/dL and no safe BLLs in children has been identified, the BLLs measured in this study continue to constitute a major public health concern for Kinshasa. The emphasis should shift to examine the contributions of non-gasoline sources to children’s BLLs: car batteries recycling in certain residences, the traditional use of fired clay for the treatment of gastritis by pregnant women and leaded paint.

## Background

Lead is a major worldwide public health concern, given the high levels of environmental contamination and the severe and long term neurotoxic effects of lead. The level of lead in the blood is a highly reliable biological marker of recent exposure to lead. Elevated blood lead level (BLLs) (10 μg/dL or above) has been associated with toxicity in the developing brain and nervous system of young children, leading to lower intelligence quotient (IQ) [1-3]. According to recent evidence, however, loss of IQ was observed in children with blood lead levels below 10 μg/dL [4-9], so prevention activities should be initiated to bring down the levels of lead in the blood to the lowest possible level. Measures to reduce and control lead use and prevent human exposure to lead, in particular for children, have been put in place at national and international levels [2,10].

Lead-containing gasoline remains the most important source of atmospheric lead and is a significant contributor to the lead burden in the body. The phasing out of lead from gasoline, first in United States of America (USA), has resulted in a significant decrease in BLLs in children during the last two decades. In the USA and Europe, epidemiologic evidence has driven the successful removal of lead from gasoline and paint, resulting in average BLLs in the USA today that are one-tenth what they were in the late 1970s: the prevalence of BLLs among children decreased from 8.6% in 1988–1991 to 1.4% in 1999–2004 which is an 84% decline [11]. Predictable reductions in BLLs have been described in other countries: in the Belgian population, the median values of blood lead concentration were 17 in 1978 and 7.8 μg/dL eleven years later, i.e., a lowering of about 55% [12]. In Helsinki, the mean concentration of lead in the blood of children in day-care centers was 4.6 μg/dL in 1983 and 3.0 μg/dL five years later [13]. However, in much of the world such as African nations, progress to reduce exposure to this toxic element has been slower. In 2002, Sudan was the only one of the 48 Sub-Saharan countries to entirely use unleaded gasoline. In May 2004, more than half of the gasoline sold in Sub-Saharan Africa was unleaded. On December the 27th, 2005, the United Nations Environment Program (UNEP) declared that the promise made to free Sub-Saharan Africa from leaded gasoline had just been fulfilled, and that the campaign in favor of suppression of lead in fuel was on the way to become a real success story in the developing world. In 2009, ongoing efforts by African countries at the UNEP to eliminate lead from gasoline are to be lauded, because they have greatly improved the cognitive potential of future generations of children [14].

In the DRC (Democratic Republic of Congo), although there are multiple sources of lead exposure, leaded gasoline is a common high dose source of exposure for children living in Kinshasa: surveys conducted in 2004 and 2008 shown that the prevalence of elevated BLLs was 63% and 71% respectively [15,16] that indicates a public health issue which requires corrective actions. A process of phasing out leaded gasoline started in December 2005 (Declarations of Dakar in 2001 and Johannesburg in 2002) and completed in December 2009 in DRC. A short visit in December 2010 to the various gas stations of Kinshasa city indicated that there are no gas pumps with leaded gasoline (data not shown). The present study originated from that observation and its main objective was to test for reduction in pediatric BLLs in Kinshasa, DRC, by comparing BLLs collected in 2011 (2 years after use of leaded gasoline was phased out in Kinshasa) to those collected in surveys conducted in 2004 and 2008 (when leaded gasoline was still used in Kinshasa).

## Methods

In the absence of reliable population registers and in view of the practical difficulties of conducting a truly random sampling in the population of Kinshasa, we applied a two-stage systematic sampling approach [17]. In the first stage, the 22 administrative entities of Kinshasa were listed in alphabetical order and 11 out of them were selected as follows: a first entity was drawn randomly from the list and every other subsequent entity was then included, thus ensuring a comprehensive coverage of the entire urban area of Kinshasa. In the second stage, we aimed to recruit about 10 healthy boy and girl subjects between 1 and 5 years old from each of the 11 entities. 110 parents of children were contacted by project staff to take part in this survey. All parents were informed about study; those who agreed to participate (100 parents) read and signed a letter of informed consent. 100 children provided a blood sample and were included in the present study (91% of the target number was reached). Data collection was completed during January 2011. This study was approved by the Congolese committee of medical ethics and the study results will be informed back to individual sample donors with proper explanations.

Analysis of blood lead Blood samples were collected by venipuncture using 85.1160 needles and metal free tubes (SMonovette Trace Element K2EDTA, D-51588, Numbrecht) in the local health centers after careful cleaning of the skin at the venipuncture site. The samples were then kept frozen and transported in a cool box to be analyzed by the Louvain centre for Toxicology and Applied Pharmacology (Brussels, Belgium). Great care was taken to avoid contamination during all the steps of collection, transport and analysis. In all blood samples lead was quantified by means of inductively coupled argon plasma mass spectrometry (ICP – MS) [18,19] with an Agilent 7500cx ICP-MS using a Babington nebulizer, following 1:10 dilution in a basic diluent: 1-butanol (2% w/v), EDTA (0.05% w/v), Triton X-100 (0.05% w/v), NH4OH (1% w/v), internal standards (Sc, Ge, Rh and Ir) and MilliQ water (ISO 15189 accredited method). The reagent blank–based LOD (limit of detection) for lead was employed and all values measured were greater than LOD (1 μg/dL).

### Statistical analysis

The distribution of BLL was log-normal upon visual inspection; therefore, the lead concentrations were expressed in terms of geometric means (GM) with 95% of confidence intervals and geometric standard deviation (GSD), arithmetic mean, median, , minimum (Min) and maximum (Max). We calculated Anova Fisher test, student’s t-test on log-transformed values or chi-square test for comparing subgroups according to age and sex or to BLL 2004, BLL 2008 and BLL 2011. Statistical significance was defined as p < 0.05. All statistical analyses were performed using SAS software package, version 9.2 (SAS Institute Inc., Cary, NC).

## Results

Table 1 shows that most of the children in the study were boys (64%) and although the ages ranged from 1 to 5 years, the mean age of the children was 2.9 years. Using our demographic characteristics, children in current study were not different to those tested in 2004 and 2008 in terms of age (p = 0.87) and sex (2004 vs 2008, p = 0.34) (2004 vs 2011, p = 1.68) (2008 vs 2011, p = 0.045).

**Table 1:** Comparison of socio-demographic characteristics in children from urban area of Kinshasa −2004, 2008 and 2011

		**2004**	**2008**	**2011**
N		100	55	100
Age (Mean ± SD) ^**a,b**^		3.2 ± 1.6	2.8 ± 1.3	2.9 ± 1.6
Sex ^**c**^	Boys, n (%)	55 (55%)	26 (47%)	64 (64%)
	Girls, n (%)	45 (45%)	29 (53%)	36 (36%)

^**a**^ Arithmetic mean ± Standard deviation.

^b^ Anova three -sample Fischer test, p = 0.87.

^c^ Chi-square test: (2004 vs 2008, p = 0.34) (2004 vs 2011, p = 1.68) (2008 vs 2011, p = 0.045).

The BLLs ranged from 1.5 to 22.0 μg/dL, with the GM and median levels, respectively, equaling 8.7 μg/dL and 8.6 μg/dL (Table 2). Sex was not associated with differences in BLLs [8.5 μg/dL for boys (n = 64) versus 9.1 μg/dL for girls (n = 36), p = 0.46) or in elevated BLLs [12.8 μg/dL for boys (n = 17) versus 12.7 μg/dL for girls (n = 24), p = 0.88].

**Table 2:** Mean blood lead levels of children from urban area of Kinshasa in 2011

		**N**	**GM [95% CI]**	**GSD**	**AM**	**Median**	**Min**	**Max**
All		100	8.7 [8.0-9.5]	3.7	9.5	8.6	1.5	22.0
Sex ^**a**^	Boys	64	8.5 [7.6-9.5]	3.8	9.3	8.2	2.2	22.0
	Girls	36	9.1 [7.8-10.6]	3.6	9.9	9.4	1.5	20.0

^a^ Two -sample t test: p = 0.46, *GM,* Geometric mean; *GSD,* Geometric standard deviation; *AM,* Arithmetic mean; *Min,* Minimum; *Max,* Maximum.

**Figure 1:**
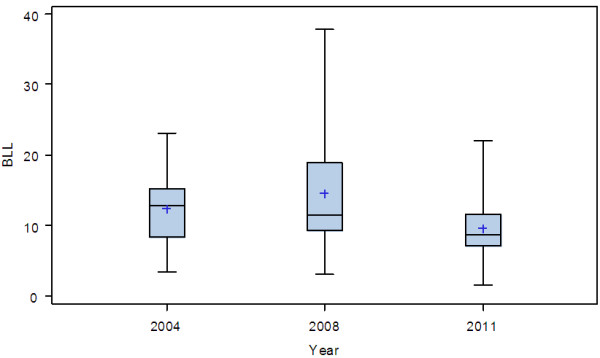
**Box plot of the distribution of BLLs (μg/dL) measured at different time period (year).** Anova Fisher test: |F| = 10.38, p <0.001.

Table 3 shows that, of the 100 children tested, 41 (41%) were found to have elevated BLLs. A smaller percentage (3%) of the children had elevated BLLs ≥ of 20 μg/dL. The proportion of children with elevated BLLs was higher in 2004 (63%) and 2008 (71%) as compared to current study, this proportion was reduced to 22% (from 2004) and 30% (from 2008) in 2011 (Table 3). The BLLs in this study were similarly reduced from 12.4 μg/dL (11.2 μg/dL) to 8.7 μg/dL, p <0.001 (Table 3; Figure 1).

**Table 3:** Comparison of blood lead levels in children from urban area of Kinshasa – 2004, 2008 and 2011

	**2004**			**2008**			**2011**		
	N	GM	%	N	GM	%	N	GM	%
		(95% CI)	≥ 10 μg/dL		(95% CI)	≥ 10 μg/dL		(95% CI)	≥ 10 μg/dL
Urban area	100	12.4	63^b^	55	11.2	71 ^b^	100	8.7	41^b^
		(11.4-13.3) ^a^			(10.3-14.4) ^a^			(8.0-9.5) ^a^	

^a^ Anova three -sample Fischer test: p < 0.001.

^b^Chi-square test: p = 0.001.

*GM,* Geometric mean.

**Figure 2:**
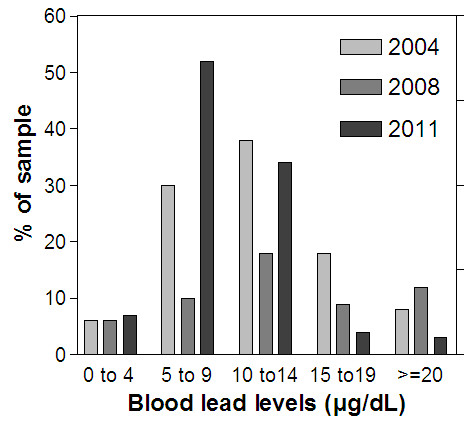
Comparison of blood lead distribution in Kinshasa (2004, 2008 and 2011).

Using a three-sample Fisher test, this reduction is statistically highly significant (|F| = 10.38, p <0.001). As can be seen from Figure 2, the blood lead distribution determined in the 2011 study is significantly reduced relative to that measured in 2004 and 2008.

## Discussion

Children can be exposed to lead from multiple sources. Because leaded gasoline was a common high dose source of exposure for children living in urban area of Kinshasa [the ambient air levels in Kinshasa, ranging from 570 to 5220 ng/m^3^ in urban area in 2008 before the total phasing out [16]], the focus of public health efforts should continue to be on phousing out exposure to leaded gasoline.

However, there are other less-common sources of lead in Kinshasa that also have high-lead content. Since 2003–2008, Tuakuila et al. [15,16] have provided valuable information on urban population’s BLLs and risk factors for elevated BLLs in Kinshasa. Other possible sources of lead exposure (GM, 95% CI) in Kinshasa include house paint chips (25 μg/g [15 – 36]), house Portland cement (15 μg/g [12 – 20]), indoor dust (720 μg/m^2 ^[555 – 934]), playing area outdoor soil (39 μg/g [22 – 67]), drinking water (0.24 μg/L [0.16 – 0.37]), fired clay use for the traditional treatment of gastritis by pregnant women (190 μg/g [142 – 255]), car batteries recycling activities in certain residences (lead in soil was 51 μg/g [15 – 181] vs 35 μg/g [18 – 64] in residences without these activities). Because these surveys are based on an urban representative sample, estimates can be generalized only to the Kinshasa urban population, the sample is not designed to provide estimates for specific groups of DRC population such as others cities, rural or industrial areas where the studies of BLLs and the risk of elevated BLLs are not known. Local surveillance data are needed to assess and manage local risks.

Regarding our study population, great care was taken to select a representative sample of the Kinshasa children. In the absence of reliable demographic data, it is not possible to assess the exact representativeness of our sample. However, there are no reasons to suspect a bias caused by self- selection based on either high or low exposure to lead.

Demographic characteristics (age and sex) of children in this study were compared to those found in the same population in 2004 and 2008 [15,16]. No significant differences were observed between the subgroup studied (Table 1), indicating that our sampling strategy (unweighted clusters) did probably not introduce a strong bias in the representativeness of our population sample.

The samples from 2004 and 2008 were analyzed in a different way (GFAAS) as those in 2011(ICP-MS). The ICP-MS method allows analyses of lead with low detection limits as compared to GFAAS method. However, analytical results are not significantly different [18-20]. Both methods can be used as a routine analytical method for the determination of lead in human blood samples [21].

Between 2004 and 2011 significant reductions in the BLLs of children from urban area of Kinshasa were observed (Table 3; Figures 1 and 2), with a consequent reduction in the proportion of children at risk of the neurobehavioral and other health and social ill effects associated with elevated lead exposure. It is highly likely that this reduction, at least in part, is associated with the introduction of unleaded gasoline since 2005 and completed in 2009. There is little evidence to indicate that factors such as lead water adduction pipes, socio economic status or industrial activities might have made a major contribution to the reductions in BLLs observed. First, while the BLLs of children were 12.4 μg/dL and 11.2 μg/dL in 2004 and 2008 respectively, lead stands at concentrations of 4 μg/L in drinking water [15] which is less than the 10 μg/L threshold set by the WHO. Second, according to PRB 2010 world population, 80% of population in DRC live under 2 dollars per day. Third, there are no industry releasing significant amounts of lead nor landfills were located near the study places [22].

The reductions in BLLs observed among children in this study are broadly comparable with what observed in several African countries [14]. Data suggest that following the phase-out of leaded gasoline, the evidence of reduced levels is positive (12.4 μg/dL to 8.7 μg/dL), but many children still have levels that may harm their health. This may affect their neurobehavioral performance [1,4,8,23]. Lead poisoning remains also highly prevalent among children in others African cities. For example, the BLLs were 6.4 μg/dL in South Africa [24] and 7.15 μg/dL in Uganda [25] after at least 4 years of the phase out of leaded gasoline. In 2007–2008 an investigation into the deaths of eighteen children living on the periphery of the City of Dakar, Senegal, showed severe lead poisoning from recycling of lead batteries in many households as the cause [26].

The prevalence of elevated BLLs in current study (41%) decreased dramatically as it was in 2004 (63%) and 2008 (71%), probably because of the DRC has recently completed in 2009 the phase out of lead in gasoline: a 30% decrease 2 years after the phase out of lead in gasoline began compared with studies the same population before the phase out [15,16]. Predictable reductions in BLLs have been described in several countries [12,13]. However, the prevalence of elevated BBLs in Kinshasa remains higher as compared with other African nations: 10% in South Africa [24] and 20.2% in Uganda [25], and continue to constitute a major public health concern, especially because of three insights. First, about 35% of children in current study had BLLs between 10 to 14 μg/dL (Figure 2), as it is known, the severity of signs and symptoms of lead poisoning increases with exposure [27]. Second is the special susceptibility of children, even relatively low levels of exposure: lead can cause serious and in some cases, irreversible neurologic damage, leading to permanent intellectual impairment [28]. Third, BLLs < 10 μg/dL have been associated with cognitive impairment and recent evidence suggests that there may be no safe level [3-9,23].

## Conclusions

These results demonstrate a significant success of the public health system in Kinshasa, DRC achieved by the removal of lead from gasoline. However, with increasing evidence that adverse health effects occur at BLLs < 10 μg/dL and no safe BLLs in children has been identified, the BLLs measured in this study continue to constitute a major public health concern for Kinshasa. The emphasis should shift to examine the contributions of non-gasoline sources to children’s BLLs: car batteries recycling in certain residences, the traditional use of fired clay for the treatment of gastritis by pregnant women and paint leaded.

## Competing interests

The authors declare that they have no completing interests.

## Authors’ contributions

JT drafted the manuscript. All authors commented the draft versions. All authors read and approved the final manuscript.
